# Osteonecrosis of the Hip Misdiagnosed as Lumbar-Disc Herniation: Тhree Case Studies

**DOI:** 10.7759/cureus.51730

**Published:** 2024-01-05

**Authors:** Plamen N Penchev, Petar-Preslav Petrov, Edvin Vasvi

**Affiliations:** 1 Department of Medicine, Plovdiv Medical University, Plovdiv, BGR; 2 Department of Anatomy, Plovdiv Medical University, Plovdiv, BGR; 3 Department of Neurological Surgery, Acibadem City Clinic, Varna, BGR

**Keywords:** mri, hip joint, disc herniation, relevant physical examination, hip avascular necrosis

## Abstract

Disc herniation and hip-joint pathology may present with overlapping symptoms, complicating the diagnosis and treatment strategy in some cases. To ensure a correct diagnosis, this study emphasizes the need for imaging methods like MRI scans of the hip joints, complementary to the lumbar spine, when in doubt of coexisting hip pathology with symptomatic lumbar disc herniation. A typical complaint in clinical practice among patients with lumbar disc herniation is chronic back pain, often radiating down the legs. Although there could be considerable overlap in pain between hip joint issues and disc herniation, the etiology of these two conditions might differ. In these situations, a comprehensive diagnostic evaluation is crucial, as demonstrated by the three clinical case studies provided here. This article underscores the importance of conducting thorough imaging tests such as hip-joint and spine MRI scans to accurately differentiate among various disorders. Pathologies such as avascular necrosis can go unnoticed on X-rays of the hip joint, but an MRI scan provides a more precise diagnosis in these situations. The cases described here highlight the challenge of differentiating between hip-joint pathology and disc herniation due to their similar symptoms. For a diagnosis to be made quickly and accurately, modern imaging techniques must be used in conjunction with a comprehensive diagnostic approach and physical examination, which will improve patient outcomes and enable proper management.

## Introduction

Hip-joint and lumbar spine pain can result in significant disability. Lower back pain associated with leg pain is the most common consequence for patients with both hip and lumbar spine pathologies [1]. The primary source of pain can be misdiagnosed due to a lack of physical examination, leading to inappropriate treatment. Delayed diagnosis or misdiagnosis of hip pathology can significantly impair a patient's quality of life. This can be prevented by implementing an examination that looks for radicular symptoms and provokes symptoms for hip pain. If hip pathology is suspected in young individuals, performing an MRI is appropriate. In 1983, the phrase “hip-spine syndrome” was first used by Offierski and MacNab, who categorized the syndrome as secondary, complex, simple, or misdiagnosed. Despite coexisting hip and lumbar spine symptoms, the primary source of disease is evident in patients with simple hip-spine syndrome. However, in patients with complex hip-spine syndrome, despite a thorough physical examination, the exact cause of the symptoms remains unknown, necessitating injectable diagnostics and additional diagnostic testing. The symptoms in one region are secondary to the primary pathology in patients with secondary hip-spine syndrome when both illnesses are interdependent [1, 2]. We present three cases of symptomatic lumbar disc herniation with coexisting avascular necrosis of the hip joint, which is the leading cause of the leg pain.

## Case presentation

Case 1

History

A 60-year-old female patient reported back pain and groin pain in the left leg. The pain was more severe in the leg than in the back. The onset of symptoms was 30 days prior to the clinical examination by a neurosurgeon and occurred suddenly after getting up from the bed. The patient took NSAIDs and PPI for 30 days, experiencing an 80%-90% reduction in pain. The medications were 400 mg of Etodolac taken orally twice a day and 30 mg of Lansoprazole taken orally once a day. The pain limited the patient's ability to continue work as a saleswoman and her workout routine. She sought consultation with a neurosurgeon due to the return of pain in the left leg after stopping the medication.

Physical Examination

**Figure 1:**
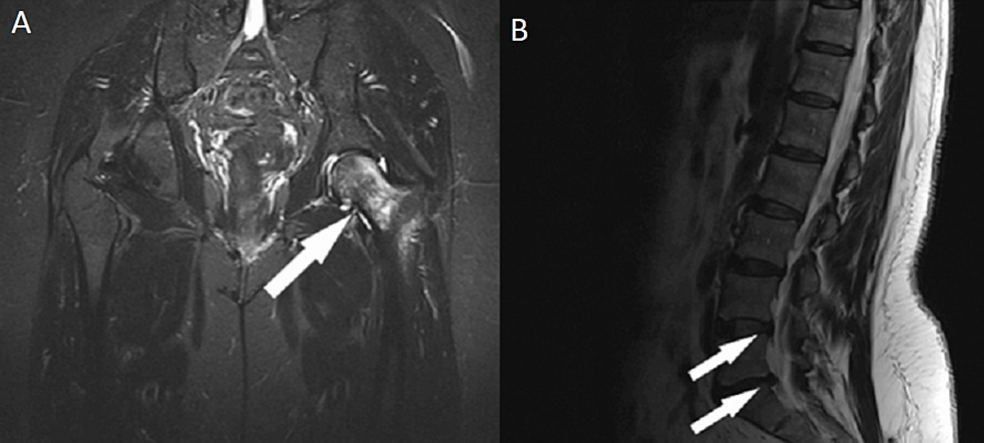
MRI: (A) Coronal plane, findings of avascular osteonecrosis of the left femoral head, and (B) sagittal plane, asymptomatic disc herniations at L4-L5 and L5-S1

Clinical examination revealed that the groin pain was more severe than the back pain. The patient presented with left peroneal paresis M4/5 of one-month duration, left sacroiliac pain, and a positive left FABER test. A CT scan on February 3, 2020, found degenerative changes in the lumbar spine; an MRI of the hip joints and lumbar region on October 25, 2022, revealed asymptomatic disc herniations at L5-S1 and L4-L5, as well as evidence of avascular necrosis of the left hip joint (Figure 1). The patient was referred to an orthopedist for consultation and further treatment of the hip pathology.

Diagnosis and Treatment

This case illustrates the possible coexistence of spine pathology and hip pathology, which can mimic the source of leg pain. Clinical examination is crucial for differentiating the etiology of the pain (radiculopathy from joint pain). Based on the findings during the clinical examination, the treating physician can order the most appropriate imaging methods to accurately diagnose the condition. The patient was referred to an orthopedist for the treatment of avascular necrosis of the hip joint.

Postoperative Follow-up

Following a surgical procedure to treat avascular necrosis of the left hip joint, the patient underwent extensive rehabilitation. The goals of physical therapy were to strengthen the muscles surrounding the hip joint, increase the range of motion, and gradually resume weight-bearing activities. The patient's pain level decreased, and her mobility gradually improved. By the third month following surgery, she reported much less pain in the left hip and groin area. An MRI six months after the initial surgery revealed that the hip joint had recovered favorably, with no indications of postoperative complications or recurrent avascular necrosis. At the one-year follow-up, the patient's left hip joint had nearly recovered, and she had resumed daily activities with no significant limitations or pain.

Case 2

History

A 54-year-old male patient had experienced back pain radiating to his left leg since November 2022. The patient had managed his pain with oral NSAIDs, resulting in a significant improvement in symptoms. He experienced pain in the area of the left inguinal fold and on the back surface of his left leg. The patient consulted with a doctor, who referred him for an imaging diagnosis of the lumbar spine, which revealed lumbar disc herniation at L5-S1. When the patient stopped the NSAIDs medication, the pain in the groin area returned, prompting him to seek a second opinion from a neurosurgeon four months after the onset of the pain.

Physical Examination

**Figure 2:**
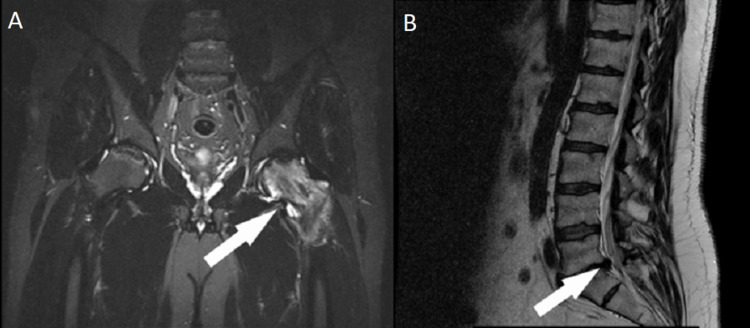
MRI: (A) Coronal plane, MRI showing avascular osteonecrosis of the left femoral head, and (B) sagittal plane, MRI showing disc herniation at L5-S1

Upon clinical examination with the neurosurgeon, the left FABER test was positive, and there was radiculopathy S1 on the left side. It was found that the patient had more pain in the left hip joint than in the S1 radiculopathy. An MRI of the lumbar area revealed disc herniation, but since the pain in the groin area was more severe, the neurosurgeon ordered an MRI scan of the hip joint which revealed avascular necrosis of the left hip joint (Figure 2).

Diagnosis and Treatment

The patient was referred for a follow-up consultation with an orthopedist for the hip pathology, and the lumbar disc herniation was only observed because the pain from the hip pathology was the main symptom. This case demonstrates that there can be a coexistence between radicular symptomatology from lumbar disc herniation and pain in the area of the hip joint from avascular necrosis of the same. Clinical examination and imaging are of primary importance in establishing the diagnosis and the origin of the pain. Lumbar disc herniations can be managed conservatively in 90% of cases with a successful outcome and improved quality of life.

Postoperative Follow-up

The avascular necrosis of the left hip joint was surgically treated, followed by a rehabilitation program. The patient adhered to the postoperative rehabilitation plan, focusing on exercises for flexibility and strength. Within four months of the procedure, the patient reported significant improvement in hip joint mobility and decreased left groin pain. Eight months after the successful recovery of the hip joint, a follow-up MRI scan revealed no complications or recurrence of avascular necrosis. The patient exhibited excellent functional outcomes at the one-year follow-up, with no discomfort during physical activities and an improved range of motion.

Case 3

History

A 45-year-old male patient experienced very severe pain in his left leg for 18 months. He visited multiple doctors and was conservatively treated with oral NSAIDs for lumbar disc herniation (which was not confirmed with diagnostic imaging) such as ibuprofen, etodolac, and flurbiprofen orally with maximum dosage parenterally for weeks without effect. However, no physical examination was performed on the patient, and he was not advised to undergo an MRI of the hip joints. During routine lab tests, it was discovered that he had a C-reactive protein value of 50. During this period of pain, the patient could not practice his profession due to reduced quality of life.

Physical Examination

The patient sought consultation with a neurosurgeon, who found painful performance of the FABER test with the left leg and negative Straight Leg Raise (SLR) with the left leg. Postural observation revealed a shortening of the left leg by 2 cm compared to the right after measurement. Movement testing revealed increased pain with flexion of the left leg and a significantly decreased range of motion due to pain.

Diagnosis and Treatment

**Figure 3:**
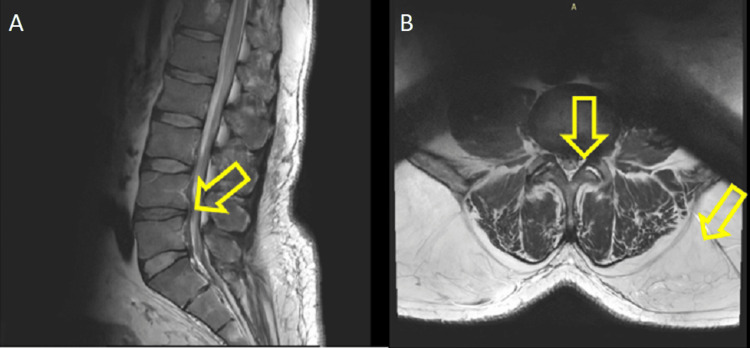
MRI: (A) Sagittal plane, MRI of the lumbar spine discovered dehydration and degeneration of the disc of L4-L5, narrowing of the disc space of L4-L5, and (B) axial plane, L4-L5 foraminal disc herniation

**Figure 4:**
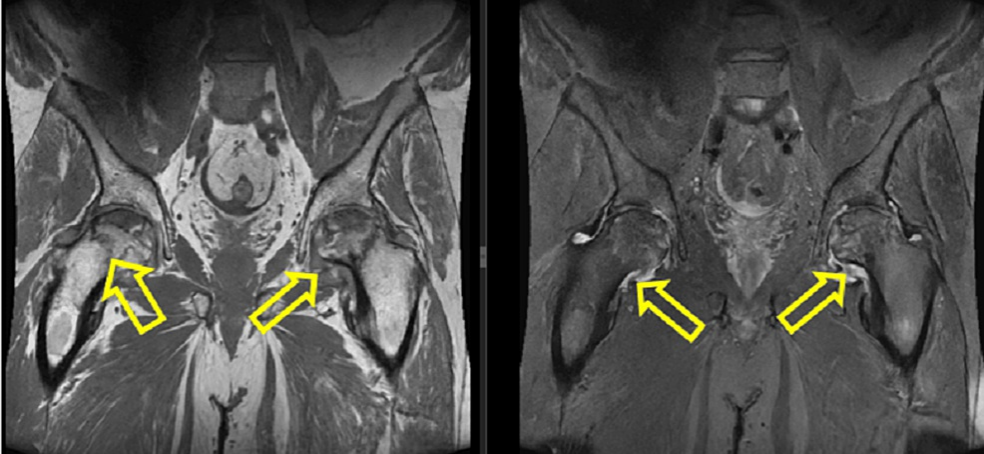
MRI, coronal planes. MRI of the hip joints discovered bilateral avascular necrosis of the hip joints

**Figure 5:**
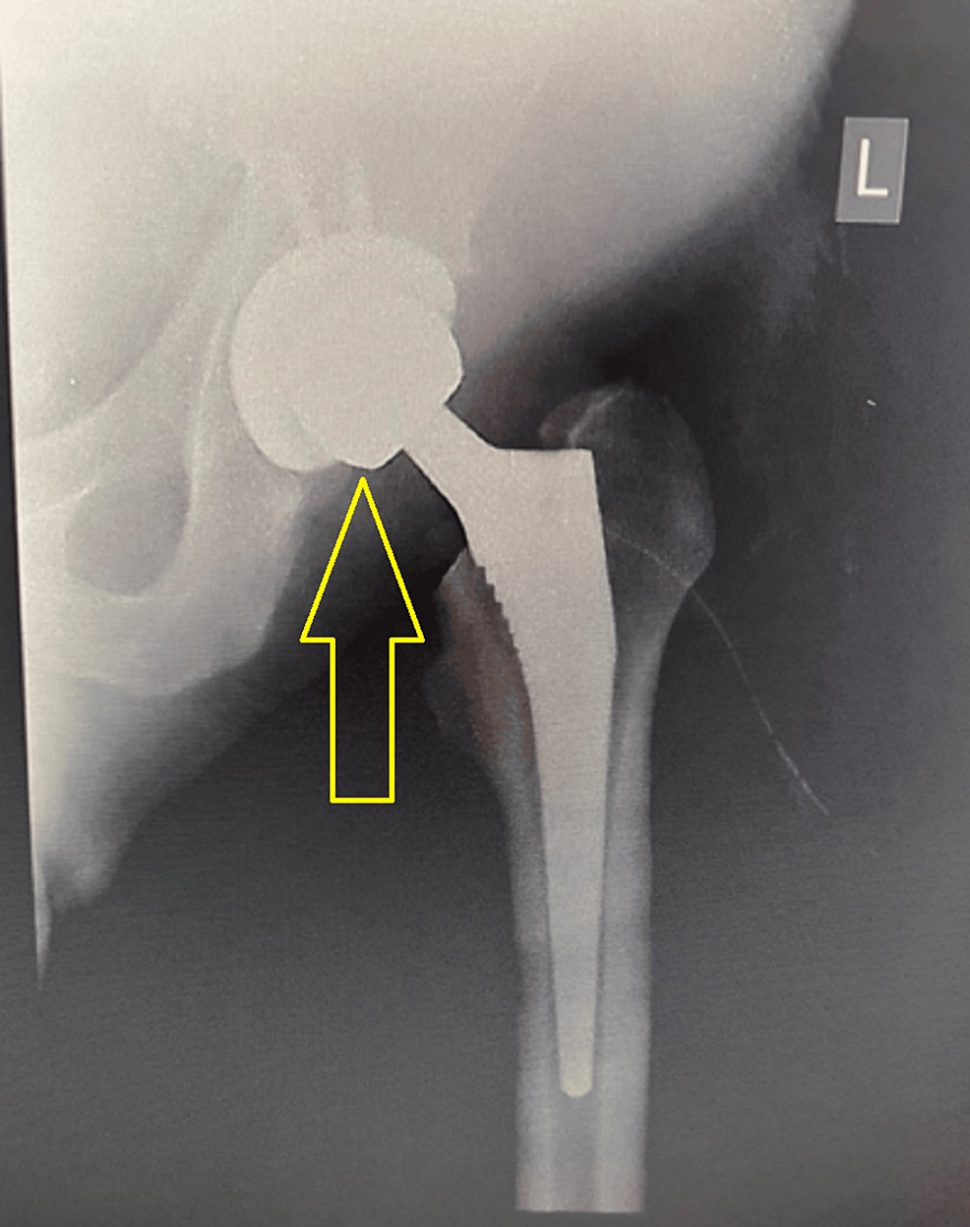
Postoperative X-ray of the left hip joint, coronal plane. X-ray showed successful hip replacement and resolution of symptoms

An MRI of the lumbar spine revealed L4-L5 asymptomatic foraminal disc herniation (Figure 3). Therefore, the neurosurgeon ordered an MRI of the hip joints because the groin pain could not originate from an L4-L5 disc herniation. The MRI of the hip joints discovered bilateral avascular necrosis of the hip joints (Figure 4). The patient was referred to an orthopedist for surgical treatment of the symptomatic avascular necrosis in the left leg (Figure 5). Postoperatively, the patient is doing well, with an improved quality of life and no pain in the left leg. He has returned to work. This case reveals that high CRP values with persistent leg pain could be indicative of hip pathology and that the source of the pain should be sought aggressively if conservative treatment doesn’t improve the quality of life.

Postoperative Follow-up

To assist in recovery, the patient underwent a structured rehabilitation program after surgery for symptomatic avascular necrosis in the left leg. The focus was on improving mobility and re-establishing hip joint function. The patient reported significant improvement in left hip joint mobility and decreased leg pain within three months of the surgery. Six months after the initial surgery, a follow-up MRI scan showed that the avascular necrosis in the left hip joint had successfully recovered, with no signs of complications. The patient's functional status had significantly improved at the one-year follow-up, reporting a return to good activity levels and no discomfort or pain when engaging in physical activities.

## Discussion

A clinical history should begin by discovering the duration, severity, location, and character of the patient’s pain, as well as determining whether the pain is present during activity, at rest, or both. A thorough hip and spinal evaluation, along with gait analysis, inspection, and palpation, should all be part of the clinical examination for a patient who may have pathologies of the lumbar spine and hips. Range-of-motion hip exercises are necessary to assess for hip pathology, which is indicated by pain at the terminal range of motion and loss of internal rotation [1-5]. However, 71% of patients with hip pathology have reported having buttock pain and pain distal to the knee [6]. According to Lesher et al., 55%-57% of patients with hip pathology have reported experiencing groin and thigh pain [7]. According to Brown et al., patients with radiculopathy brought on by lumbar spine pathology are 14 times more likely to have limited internal rotation and are seven times more likely to report groin pain and a limp [3]. Positive tests that reveal whether the symptoms are caused by lumbar spine or hip pathology include the femoral nerve stretch, contralateral straight leg raise, and straight leg raise. The snapping iliopsoas test, the FABER test, the FADIR test, and instability tests are additional positive provocative tests that can help explain hip pathology [8-10].

The use of modern imaging methods such as MRI and CT scans of both hip joints and the lumbar spine is strongly recommended, because not using these can lead to misidentification of the source of the pain, which can lead to misdiagnosis and inadequate treatment [11, 12]. The differential diagnoses for patients with hip-spine syndrome include hip osteoarthritis, avascular osteonecrosis of the hip joint, or lumbar radiculopathy [13-16]. Patients with symptomatic lumbar disc herniations can be successfully treated conservatively in 90% of cases. Patients who need surgical treatment for lumbar disc herniations are those who have severe radiculopathy and severe motor weakness, which does not improve with conservative treatment for at least 6 weeks. Asymptomatic lumbar disc herniations do not need any kind of treatment. If the patient has groin pain and decreased range of motion, it should raise a flag for the treating physician for possible hip-joint pathology, which should be addressed properly with imaging methods.

All the patients in our study had lumbar disc herniation with hip-joint pathology which are pathologies that could cause leg pain, but the leading source of the pain in these cases was the hip joint, not the radiculopathy. Misdiagnosis could lead to psychological issues, impaired quality of life, delayed appropriate treatment, risk of adverse drug reactions due to increased intake of medication, and inability to perform physical labor.

Due to the inherent limitations of a case series, a cause-and-effect relationship cannot be inferred. However, the intention of this series is to describe the clinical reasoning process of clinicians when deciding how to manage patients who have leg pain and possible coexisting pathologies in the lumbar spine and hip joint. It has been demonstrated that patients receiving MRI of both the lumbar spine and hip joint and detailed physical examination have a better outcome and improved quality of life.

## Conclusions

To determine the etiology of pain in patients exhibiting clinical manifestations of back pain associated with lower extremity pain, a thorough clinical examination and a systematic and detailed patient history are necessary. Modern imaging techniques like CT and MRI scans of the spine and hip joints are advised for accurate diagnosis because of their high sensitivity and specificity, helping to further define the primary source of symptomatology and directing the proper course of treatment. In cases where a secondary cause of symptoms manifests, treatment may be necessary even though the primary pathology is managed. Certain patients may be more likely to receive a misdiagnosis due to corticosteroid and NSAIDs abuse, which will delay making the correct diagnosis.

The three clinical cases highlight the complex challenges of identifying and treating hip-joint pathology that coexists with asymptomatic lumbar disc herniation. Each case demonstrates the importance of thorough clinical examinations in combination with modern imaging methods to identify the various causes of leg pain. The significant difference between these pathologies is revealed in the cases highlighted by the essential discovery of avascular necrosis within the hip joint, which was initially obscured by symptoms of lumbar disc herniation. The postoperative outcomes, which demonstrate impressive recuperation after specific surgical interventions, validate the life-changing influence of precise diagnosis and immediate intervention. The clinical cases illustrate the need for individualized patient care, highlighting the essential role of a multidisciplinary team that includes neurosurgeons, radiologists, and orthopedic specialists.
